# Dynamic Interactions Between Mega Symbiosis ICEs and Bacterial Chromosomes Maintain Genome Architecture

**DOI:** 10.1093/gbe/evac078

**Published:** 2022-05-26

**Authors:** Alexandra J. Weisberg, Joel L. Sachs, Jeff H. Chang

**Affiliations:** Department of Botany and Plant Pathology, Oregon State University, Corvallis, OR 97331, USA; Department of Evolution Ecology and Organismal Biology, University of California Riverside, Riverside, CA 92521, USA; Department of Microbiology and Plant Pathology, University of California Riverside, Riverside, CA 92521, USA; Institute for Integrative Genome Biology, University of California Riverside, Riverside, CA 92521, USA; Department of Botany and Plant Pathology, Oregon State University, Corvallis, OR 97331, USA

**Keywords:** evolution, horizontal gene transfer, symbiotic nitrogen fixation, mobile genetic elements, integrative and conjugative elements

## Abstract

Acquisition of mobile genetic elements can confer novel traits to bacteria. Some integrative and conjugative elements confer upon members of *Bradyrhizobium* the capacity to fix nitrogen in symbiosis with legumes. These so-called symbiosis integrative conjugative elements (symICEs) can be extremely large and vary as monopartite and polypartite configurations within chromosomes of related strains. These features are predicted to impose fitness costs and have defied explanation. Here, we show that chromosome architecture is largely conserved despite diversity in genome composition, variations in locations of attachment sites recognized by integrases of symICEs, and differences in large-scale chromosomal changes that occur upon integration. Conversely, many simulated nonnative chromosome–symICE combinations are predicted to result in lethal deletions or disruptions to architecture. Findings suggest that there is compatibility between chromosomes and symICEs. We hypothesize that the size and structural flexibility of symICEs are important for generating combinations that maintain chromosome architecture across a genus of nitrogen-fixing bacteria with diverse and dynamic genomes.

**Significance**

It has been hypothesized that symbiosis integrative conjugative elements (symICEs) that confer symbiotic nitrogen fixation and with a polypartite configuration have selective advantages over those that have a monopartite configuration. In this study, symICEs of *Bradyrhizobium* were characterized in a phylogenetic and genomic context and findings suggested that polypartite and monopartite symICE configurations can cause fitness costs in nonnative combinations and the advantages conferred by either are realized only in compatible chromosomes. Therefore, evidence suggests that no symICE configuration is generally more advantageous than the other and supports a hypothesis that selective advantages lie in the flexibility of symICEs in adopting different configurations and recombining in different regions of chromosomes.

## Introduction

Horizontal gene transfer (HGT) drives genome diversification and innovation. ICEs are agents of HGT that can transfer between bacterial cells and facilitate recombination with chromosomes (Johnson and Grossman 2015). Critical to their role in the evolution of bacteria, ICEs often have cargo genes that confer fitness benefits, such as nitrogen-fixing symbiosis. Despite their importance and widespread nature, evolution of ICEs and their adaptations to bacterial chromosomes are poorly understood (Botelho and Schulenburg 2021).

Genome organization constrains diversification (Baltrus 2013). Bacterial chromosomes are replicated bidirectionally whereby two replication forks initiate and diverge from an origin of replication (ori) and in circular molecules, stop within a terminator (ter) region. The two chromosome halves (replichores) are typically balanced in size to coordinate timing of replication. Replichores also frequently exhibit a bias in the orientation of coding sequences (CDSs), predicted to reduce head-on collisions between DNA and RNA polymerases, and asymmetry in the composition of nucleotides (Rocha 2004). Large-scale genomic rearrangements and horizontally acquired elements that upset chromosomal architecture can have severe fitness effects (Hill and Gray 1988; French 1992; Liu et al. 2006; Esnault et al. 2007; Darling et al. 2008; Srivatsan et al. 2010). However, horizontally acquired elements tend to be concentrated at recombination hotspots, suggesting that these elements and bacterial chromosomes coevolved to accommodate acquisition, whereas preserving architecture (Oliveira et al. 2017).

The *Bradyrhizobium* genus has a large and diverse accessory genome (Weisberg et al. 2022). The symICEs of these bacteria have clusters of genes that confer host specificity and nitrogen fixation, respectively (Poole et al. 2018). These symICEs are mega sized (typically between half to one megabase in size) because they include extensive variable regions with genes not known to be necessary for symbiosis (Weisberg et al. 2022). SymICEs integrate at different tRNA attachment (*att*) sites and vary in their structural configurations as monopartite or polypartite elements. When integrated in a chromosome, elements of a polypartite symICE are located in different regions and mediate large-scale rearrangements during recombination (Haskett et al. 2016). In *Bradyrhizobium*, the most prevalent type is a bipartite tRNA-Val symICE (Weisberg et al. 2022). The large element of the bipartite tRNA-Val symICE (∼0.50 Mb) is integrated near a tRNA-Val gene and the small element (∼0.05 Mb) is integrated near *ybgC*. The monopartite symICEs of *Bradyrhizobium* are less common and integrate at one of four tRNA loci, tRNA-Ile (∼0.63 Mb), tRNA-Glu (∼0.77 Mb), tRNA-Gln (∼0.68 Mb), and tRNA-Pro (0.33 Mb) symICEs.

Acquisition of a symICE is expected to disrupt chromosome architecture. Polypartite symICEs are perplexing because they cause large inversions to chromosomes during recombination and can cause lethal deletions if the excision of elements is not executed in the proper order (Haskett et al. 2018; Weisberg et al. 2022). Although costs for polypartite symICEs have been uncovered, benefits are less clear. One set of hypotheses was posed predicting that the capacity for polypartite symICE to integrate its elements at multiple sites provides a selective advantage because of greater flexibility, stability, and opportunities for accretion than those of monopartite symICEs (Haskett et al. 2017). However, an alternative hypothesis emerged when large datasets of symICE sequences from diverse strains of *Bradyrhizobium* and *Mesorhizobium* were examined in a phylogenomic context (Greenlon et al. 2019; Weisberg et al. 2022). In both genera, the monopartite and polypartite symICEs exhibited different phylogenetic patterns, and led to the alternative hypothesis that chromosomes are adapted to a type of symICE configuration.

Here, we reanalyzed a subset of a previously generated dataset to determine variation in the organization of *Bradyrhizobium* chromosomes. Findings are consistent with the alternative hypothesis and additionally suggest that the capacity for symICEs to generate variations in conformation and attachment sites is important for them to adapt to chromosomes diverse in organization.

## Results

### Chromosome Architecture is Conserved in *Bradyrhizobium*

**Fig. 1.**
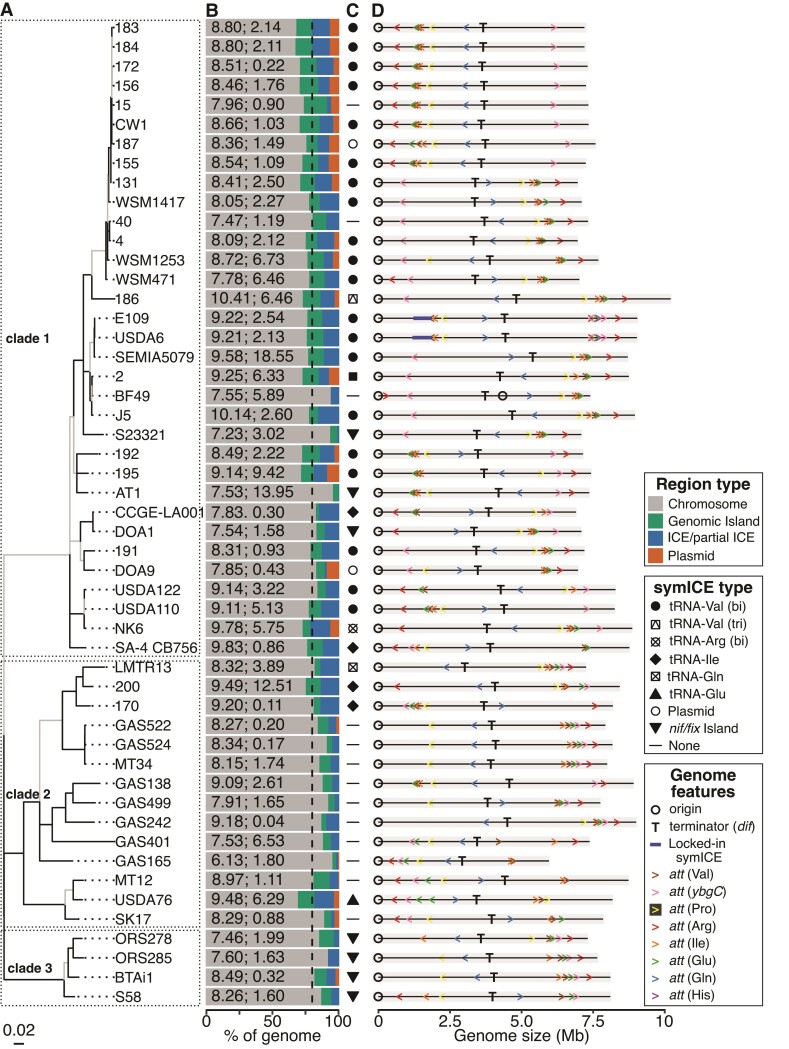
*Bradyrhizobium* genomes are diverse and balanced. (*A*) Multi-locus sequence analysis maximum likelihood (ML) tree of *Bradyrhizobium* generated based on 30 concatenated gene sequences. The tree is midpoint rooted. Branches colored black exceeded 50% bootstrap support. (*B*) Stacked bar charts showing the percentage of each native genome predicted to be genomic islands, ICEs and partial ICEs, or plasmids. Numbers listed within each bar indicate sizes of native genomes (Mb) and percent replichore imbalance. The dashed vertical line indicates an average of 21.8% of genome regions with signatures indicative of HGT. (*C*) Type of symICE. (*D*) Linear representation of chromosomes with locations and orientations of *att* sites indicated. Chromosomes natively with a symICE, but not locked in, are shown simulated with an excised symICE.

We focused on 51 *Bradyrhizobium* strains with finished or hybrid-assembled sequences (supplementary table S1, Supplementary Material online). The strains are diverse and were isolated from soils and various species of legume hosts (Weisberg et al. 2022). The strains formed three clades and their genomes are diverse in size (6.1–10.4 Mb) and composition and with an estimated average of 21.8% (4.7–32.8%) of each genome being gained horizontally, based on predictions of ICEs and plasmids (fig. 1*A* and *B*; Weisberg et al. 2022). Strains in clade 1 are enriched for bipartite tRNA-Val symICEs though there are some exceptions (fig. 1*C*). For example, strain NK6 was reanalyzed and is predicted to carry a bipartite tRNA-Arg symICE with elements near tRNA-Arg and *ybgC*. Four strains in clade 2 have one of three monopartite symICEs, though most strains analyzed here lack a symICE. Strains of clade 3 are predicted to have a *nif/fix* island with some predicted to have remnants of symICEs (Weisberg et al. 2022).

Across strains, chromosome replichores are balanced (average imbalance of 3.20% ± 3.72%; imbalance is the deviation from a perfect balance [0] where replichores are equal in size) with only three exceeding 10% replichore imbalance (fig. 1*B*; supplementary table S1, Supplementary Material online). Except for strain BF49, genomes also show a bias in orientation of CDSs and an asymmetry in nucleotides. The chromosome of strain BF49 has an unusual structure which could be due to a rearrangement or assembly error. Nevertheless, features of chromosome architecture are generally conserved across *Bradyrhizobium* strains, despite differences in phylogeny, genome size, and composition, as well as presence/absence of symICEs that differ in size, configuration, and location of recombination (fig. 1).

### Organization of *att* Sites Vary Across *Bradyrhizobium*

**Fig. 2.**
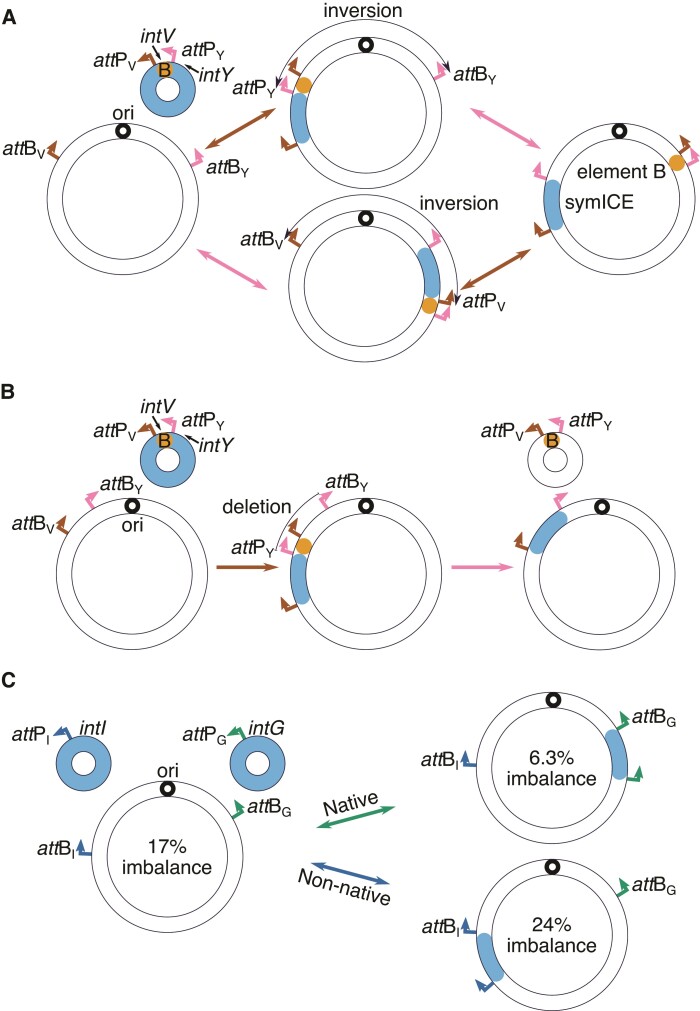
The bipartite tRNA-Val symICE causes large-scale chromosomal rearrangements. (*A*) Integration (left to right) of a bipartite tRNA-Val symICE (small orange and blue circle) in a canonical clade 1 chromosome occurs in two steps through either of two pathways. In the first step, both elements of the bipartite molecule recombine into one site. In the second step, recombination inverts the chromosome across replichores and resolves the two elements. (*B*) Integration (left to right) of a bipartite tRNA-Val symICE in a canonical clade 2/3 chromosome is predicted to also occur in two steps. In the first step, the bipartite molecule recombines into one site. In the second step, recombination causes a deletion and resolves the two elements. Recombination at the *att*B_Y_ site is predicted to have a similar outcome and was not depicted. (*C*) Integration (left to right) of monopartite tRNA-Ile (native to strain 170) or tRNA-Glu (native to USDA76) symICEs in the chromosome of strain USDA76, simulated to lack its native monopartite tRNA-Glu symICE. Calculated chromosome imbalances are shown for each combination.

Location and orientation of *att*B_V_ and *att*B_Y_ sites (attachment sites located in the tRNA-Val gene and *ybgC*, respectively) in chromosomes correlate with the phylogenetic pattern of bipartite tRNA-Val symICEs that recombine at these sequences. In 30 of 33 clade 1 strains natively lacking or simulated to lack a symICE, the two *att*B sites are in separate replichores, not equidistant from the ter region, and facing head-to-head toward the ori (fig. 1*D*). In the first integration step, a bipartite tRNA-Val symICE will increase the size of a replichore and add an *att*P site oriented head-to-head to its partner *att*B site (fig. 2*A*). In the second step, an inversion will occur that redistributes the chromosome between replichores. The amounts of symICE and chromosome involved reflect the locations of *att*P and *att*B sites and their distance apart following the first recombination step. To rebalance replichores, the size of the element that remains in the recombined replichore and the size of the element flipped to the other replichore need to offset the net change associated with the chromosome inversion. This raises the possibility that selection may favor the sizes of symICE elements and distance between *att*P_V_ and *att*P_Y_, implying that the size of the variable region is important.

**Fig. 3.**
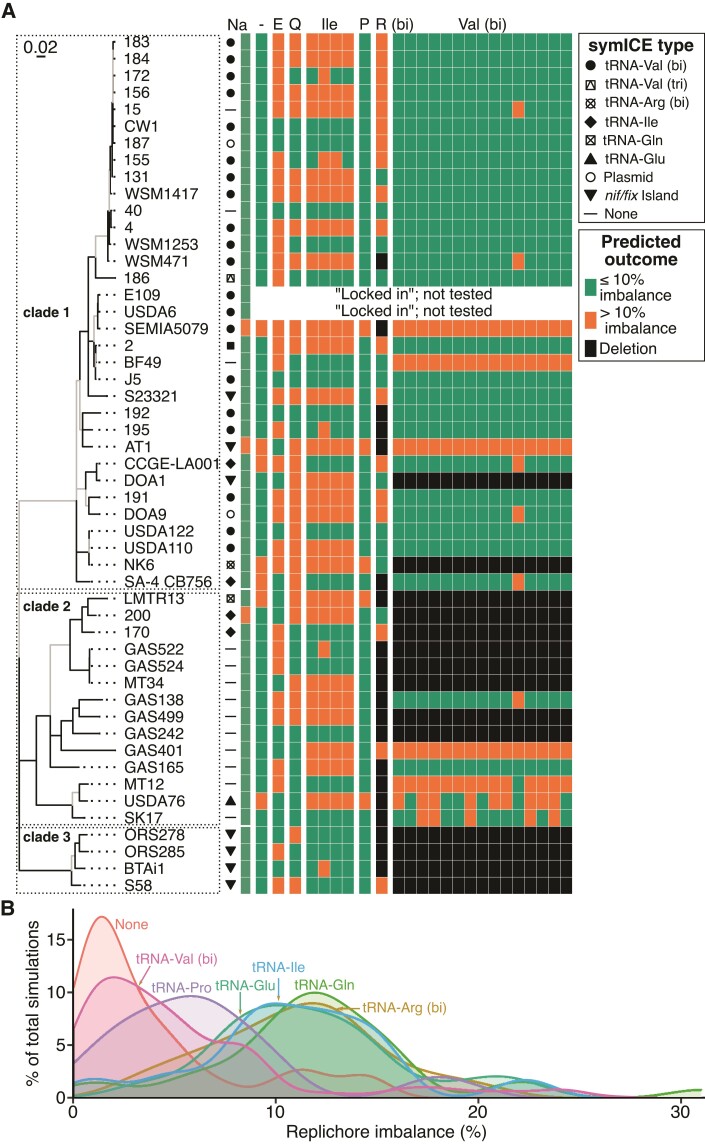
Nonnative chromosome–symICE combinations are often imbalanced or result in deletions. (*A*) Replichore imbalance of simulated chromosome–symICE combinations were categorized (≤10% imbalance [green], >10% imbalance [orange], or deletion [black]) in a heatmap and related to the MLSA ML phylogeny. Columns of the heatmap indicate: Na, native, –, no symICE, and type of symICE (E, tRNA-Glu; Q, tRNA-Gln; Ile, tRNA-Ile; P, tRNA-Pro; R, tRNA-Arg [bi]; and Val, tRNA-Val [bi]). The tree was reproduced from figure 1. (*B*) Most chromosomes of *Bradyrhizobium* strains from clade 1 are balanced in combination with a tRNA-Val symICE. Integration of symICEs and inversion of chromosomes, if appropriate, were simulated for strains in clade 1. For each type of symICE, percent of simulated combinations was plotted against percent of replichore imbalance. Values for each simulated combination are presented in supplementary table S2, Supplementary Material online. For both panels, two strains with locked-in symICEs as well as the tripartite variant of the tRNA-Val symICE were excluded from simulations.

Conversely, in 15 of 18 strains in clades 2 and 3, *att*B_V_ and *att*B_Y_ sites are located within the same replichore and arranged head-to-tail (fig. 1*D*). If a bipartite tRNA-Val symICE recombined into these chromosomes, the second step will cause an ∼1.0 Mb-sized deletion (fig. 2*B*; supplementary table S2, Supplementary Material online). The deletion is likely deleterious because many chromosomal genes, some predicted to be essential, will be lost (supplementary table S3, Supplementary Material online). A similar finding was observed in simulations with the bipartite tRNA-Arg symICE (supplementary tables S2 and S4, Supplementary Material online). Therefore, barring an unusual event, the current structures of chromosomes of most strains in clades 2 and 3, are predicted to be incapable of accommodating polypartite symICEs, providing an explanation for their observed distribution pattern in *Bradyrhizobium* (fig. 3*A*).

The locations of *att* sites targeted by monopartite symICEs are also important. Integration of a monopartite symICE occurs in one step and directly increases the size of a replichore thus requiring offsetting deletions in the same replichore or gains in the other replichore (figs. 2*C* and 3*B*). Five of six strains with monopartite symICEs have natively balanced replichores and simulated excision of their symICE resulted in an imbalance of ∼10%, consistent with changes having had occurred to balance against an increase by symICE integration (supplementary table S2, Supplementary Material online). For most strains with monopartite symICEs, chromosomes are substantially more imbalanced in at least one simulation that swapped in a nonnative type of monopartite symICE (figs. 2*C* and 3*A*; supplementary table S2, Supplementary Material online). In these cases, the *att* sites of the nonnative monopartite symICE are in a different replichore than those of the native symICEs (figs. 1*D* and 2*C*). We did not consider the effect of the monopartite tRNA-Pro symICE because its relatively small size reduces its effect on replichore imbalance (fig. 3*B*). Locations of *att* sites relative to the replichore in which offsetting large-scale chromosomal changes occur, are predicted to influence the type of monopartite symICE that will be selected for once recombined in a chromosome.

Relative to that of clade members, strains with an organization of *att* sites that deviate from common patterns are predicted to differ in their ability to acquire symICEs. The *att*B_V_ and *att*B_Y_ sites of strain DOA1 in clade 1 are organized such that a large deletion is predicted to occur upon resolution of a bipartite tRNA-Val symICE (figs. 2*B* and 3*A*; supplementary table S2, Supplementary Material online). Instead of a bipartite tRNA-Val symICE, strain DOA1 has a *nif/fix* island and a remnant of a monopartite tRNA-Ile symICE (Okubo et al. 2016; Weisberg et al. 2022). Two other strains of clade 1 each have a monopartite tRNA-Ile symICE (Weisberg et al. 2022). Simulations that swap in a bipartite tRNA-Val symICE predict a higher replichore imbalance, suggesting the distance between *att*B_V_ and *att*B_Y_ sites is not ideal in these strains (fig. 3; supplementary table S2, Supplementary Material online). Likewise, in clade 2, *att*B_V_ and *att*B_Y_ sites in strain USDA76 are arranged in a manner that accommodates a bipartite tRNA-Val symICE. But relative to the native monopartite tRNA-Glu symICE, the bipartite symICE is predicted to cause a greater imbalance in replichores (fig. 3*A*; supplementary table S2, Supplementary Material online).

The orientation of *att* sites relative to the ori can influence compatibility with symICEs because orientation determines CDS bias of introduced genes relative to the direction of replication fork movement. Across all symICEs, the orientation of CDSs is biased and exhibits asymmetry in nucleotide composition and native chromosome–symICE combinations maintain these patterns (supplementary fig. S1*A*–*H*, Supplementary Material online). In 313 of 350 possible simulations of recombination with monopartite symICEs, the SkewI index was larger, indicating greater degree of overall GC skew, when the symICE was integrated in the correct orientation relative to the *att* site versus the incorrect orientation (supplementary fig. S1*I* and table S5, Supplementary Material online). We suggest that selection has favored the orientation of *att* sites in chromosomes that maintain architecture upon symICE acquisition.

### Nonnodulating Strains are not Equivalent in their Capacity to Acquire symICEs

Nonnodulating strains potentially represent a standing pool of genetic diversity that through the acquisition of a symICE, allows the evolution of new lineages of nitrogen-fixing bacteria. However, variations in chromosomes are predicted to influence their ability to acquire a symICE. Simulated recombination of bipartite tRNA-Val symICEs in strains AT1 and BF49 of clade 1 led to predicted average replichore imbalances of 15.66% and 23.49%, respectively, suggesting selection will disfavor these strains acquiring the bipartite symICE (fig. 3*A*; supplementary table S2, Supplementary Material online). Simulations suggest at least one monopartite symICE could be gained by either strain without causing a severe imbalance in replichores, but in strain AT1, the CDS bias is predicted to be upset. In clade 2, *att*B_V_ and *att*B_Y_ sites in four strains are organized like the arrangement common among those of clade 1 and they should be capable of accommodating any symICE. Simulated recombination of strain SK17CBNU with any of the five types of symICE resulted in an estimated imbalance of 7–12% (supplementary table S2, Supplementary Material online). In contrast, in strain GAS401, two types of symICEs are predicted to cause severe imbalances ranging from 23.8% to 40.2%. Thus, simulations suggested that some nonnodulating strains can evolve a new lineage of nitrogen-fixing bacteria only if paired with a compatible symICE.

## Discussion

The dynamicity and flexibility of bacterial chromosomes yield structural diversity that in *Bradyrhizobium* impacts compatibility with symICEs but also provides the capacity to refine interactions upon recombination. We hypothesize that location, orientation, and spacing of *att* sites favor chromosome–symICE combinations that are least disruptive to overall architecture. Changes to chromosomes, induced by a polypartite symICE or independent of a symICE, can alter the chromosome to mitigate gains of large elements. Regardless of the configuration of the symICE, the accessory genome can also influence fit by shaping chromosomes. We also suggest that expansion of genome sizes by HGT of mobile genetic elements effectively diminishes the relative impact of subsequently acquired elements on replichore balance. As chromosomes increase in size, they become more insulated and have a greater capacity to capture more and larger molecules of DNA.

Detailing barriers that constrain HGT is critical for rationalizing the observed variation and understanding rules the that govern genome innovation. The advantages of symICEs are context dependent. A polypartite symICE is advantageous in strains that have *att*B sites structured to allow autonomy of the symICE to rebalance replichores, but lethal in others. Monopartite symICEs do not require conservation in relationship between multiple *att*B sites but acquisition requires a chromosome experiencing prior or subsequent large-scale changes that counter the increase in size of one replichore. Findings also explain why certain mechanisms of symICE evolution have occurred. It was previously reported that instead of a wholesale swap of symICEs, a cluster of genes from a bipartite tRNA-Val symICE recombined into a monopartite tRNA-Ile symICE, altering host specificity of four closely related strains in clade 2 (Weisberg et al. 2022). This process was favored because recombination of a bipartite tRNA-Val symICE with the chromosome of the ancestor of these strains would have caused a large and likely deleterious deletion.

Findings are predicted to be mirrored in nitrogen-fixing *Mesorhizobium* (Greenlon et al. 2019). However, we could not analyze their genomes because their degree and markers of skew were difficult to measure in those with finished genome sequences and introduced uncertainty in delimiting replichores.

In summary, because benefits are context dependent, we suggest that neither a polypartite nor a monopartite symICE is generally more advantageous than the other and hypothesize that the capacity for symICEs to vary is the advantage (Haskett et al. 2017; Weisberg et al. 2022). Their plasticity underpins evolutionary robustness to extend symbiotic nitrogen fixation in genera of bacteria with dynamic genomes.

## Materials and Methods

Previously described methods were followed to construct phylogenetic trees (Weisberg et al. 2022).

Replichores were defined based on composite skew metric (cumulative GC, AT, and CDS orientation skew) and locations of *parA* and *dif* loci (Frank and Lobry 2000; Charif and Lobry 2007). To identify the latter, the sequence, 5′-GGTGCGCATAATGTATATTATGTTAAAT-3′, of *Escherichia coli* was used as a query in blastn (BLAST v. 2.6.0) searches (Altschul et al. 1990; Blakely and Sherratt 1996). Percent replichore imbalance was calculated using the formula: absolute value of (half of the chromosome size − replichore 1 size)/half of the chromosome size × 100 (Darling et al. 2008).

A custom python script and BioPython were used to start genome sequences at the predicted ori, simulate excision of symICEs if natively in a chromosome, and induce inversions if simulating excision of a polypartite symICE integrated at *att* sites oriented head-to-head or tail-to-tail (Cock et al. 2009). Another custom script was used to simulate the integration of symICEs with symICE-free chromosomes at the corresponding *att* locus and invert regions when involving a bipartite symICE. Monopartite symICEs were simulated to integrate in both orientations relative to the *att* sites. SkewIT was used to calculate the *SkewI* index of GC skew (Lu and Salzberg 2020). For bipartite symICEs, if the two *att* sites are not oriented head-to-head or tail-to-tail, a deletion was predicted. To identify essential genes, bidirectional best BLAST searches were done using as a query a dataset of essential genes identified based on analysis of a transposon library derived from strain USDA110 (Baraquet et al. 2021).

## Supplementary Material

Supplementary data are available at *Genome Biology and Evolution* online.

## Supplementary Material

evac078_Supplementary_Data

## Data Availability

The original data underlying this article were retrieved from NCBI; BioSample numbers are listed in supplementary table S1, Supplementary Material online. Scripts underlying this article are available from: https://github.com/osuchanglab/BradyrhizobiumGenomeArchitecture.
