# Supplementary material for: Dynamic Interactions Between Mega Symbiosis ICEs and Bacterial Chromosomes Maintain Genome Architecture
Source: Genome Biol Evol. 2022 May 26;14(6):evac078. doi: 10.1093/gbe/evac078 (PMC9174649; doi:10.1093/gbe/evac078)
Supplement: evac078_Supplementary_Data [file evac078_supplementary_data.zip › SUPPLEMENTAL MATERAL.docx]

**SUPPLEMENTAL MATERAL**

**Supplemental Figure**

**Fig. S1: Cumulative skew plots. (A-G)** Plots of symICEs representative of each type, with the two elements of the tRNA-Arg symICE shown in two separate plots. **(H-I)** Plots of strain #15 (naturally lacks a symICE) simulated with a tRNA-Ile symICE recombined in two different orientations. Red line (cumulated T-A skew; black y-axis): a positive or negative slope indicates an average of more T than A bases or less T than A bases in the third codon position of predicted coding sequences, respectively. Blue line (cumulated C-G skew; black y-axis): a positive or negative slope indicates an average of more C than G bases or less C than G bases in the third codon position of predicted coding sequences, respectively. Green line (cumulated CDS skew; green y-axis): for **A-G**, a positive or negative slope indicates an excess of coding sequences oriented in the same direction; for **H-I**, the V-shaped green line with a positive slope away from the predicted ori, indicates the orientation of coding sequences generally biases transcription in the same direction as replication. Black line (cumulated combined skew; black y-axis): combines T-A and C-G and was used to predict locations for ori and ter regions.

**Supplemental Tables (available as one document with five sheets)**

**Table S1: Replichore imbalance of native *Bradyrhizobium* chromosomes**

**Table S2: Replichore imbalance of simulated chromosome-symICE combinations**

**Table S3: Genes predicted to be essential and lost upon tRNA-Val symICE-induced genome deletion**

**Table S4: Genes predicted to be essential and lost upon tRNA-Arg symICE-induced genome deletion**

**Table S5: GC skew of simulated chromosome-monopartite symICE combinations**
