# Supplementary figures and images for: Dynamic Interactions Between Mega Symbiosis ICEs and Bacterial Chromosomes Maintain Genome Architecture
Source: Genome Biol Evol. 2022 May 26;14(6):evac078. doi: 10.1093/gbe/evac078 (PMC9174649; doi:10.1093/gbe/evac078)

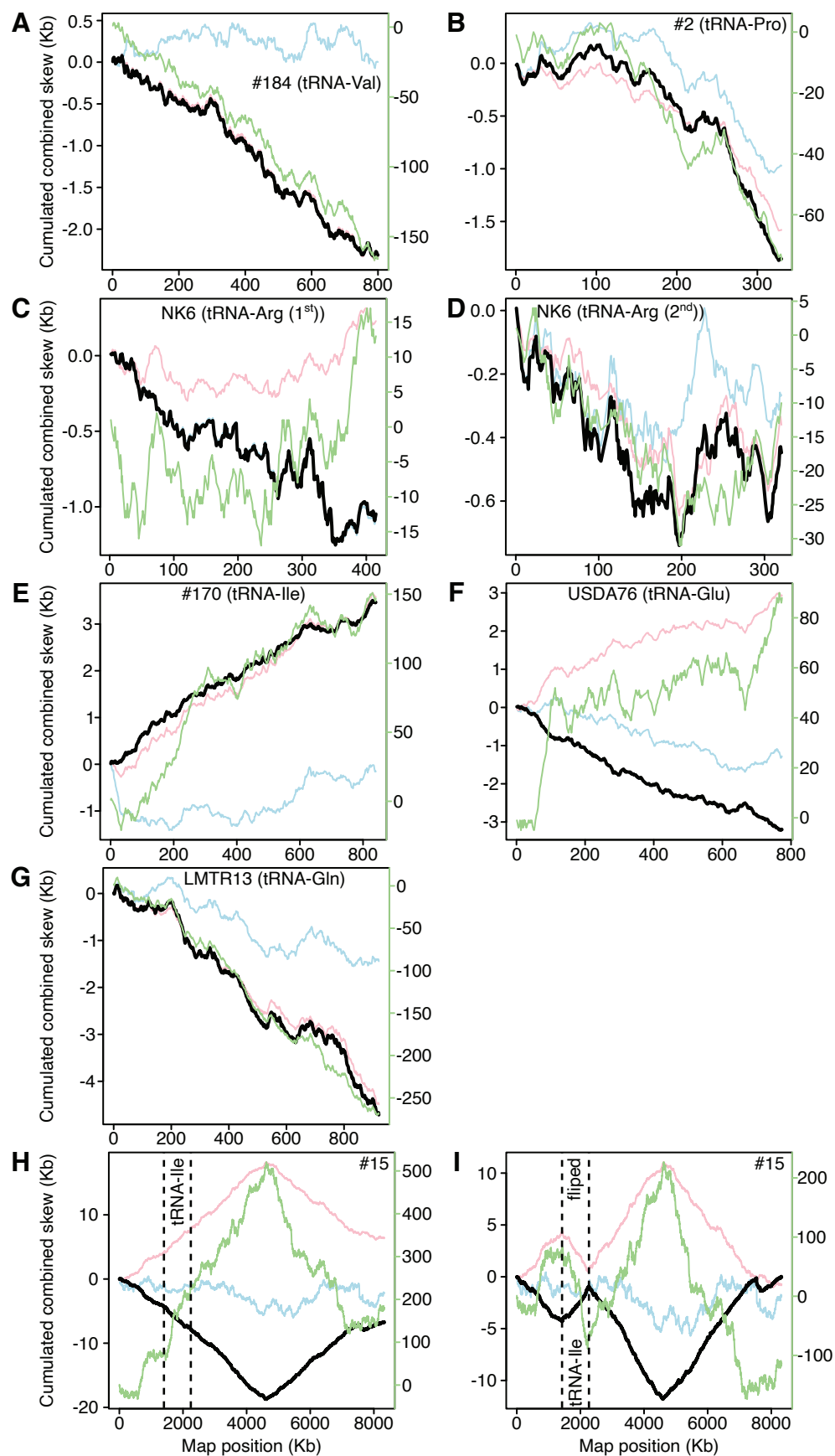

Supplement: evac078_Supplementary_Data [file evac078_supplementary_data.zip › Fig_S1_CMYK.pdf]
